# Clonal hematopoiesis and its emerging effects on cellular therapies

**DOI:** 10.1038/s41375-021-01337-8

**Published:** 2021-07-02

**Authors:** Malte von Bonin, Helena Klara Jambor, Raphael Teipel, Friedrich Stölzel, Christian Thiede, Frederik Damm, Frank Kroschinsky, Johannes Schetelig, Triantafyllos Chavakis, Martin Bornhäuser

**Affiliations:** 1 Medizinische Klinik und Poliklinik 1, Universitätsklinikum Carl Gustav Carus an der TU Dresden, Dresden, Germany; 2 AgenDix, Angewandte molekulare Diagnostik mbH, Dresden, Germany; 3 Department of Hematology, Oncology, and Tumor Immunology, Charitè-Universitätsmedizin Berlin, Corporate Member of Freie Universität Berlin, Humboldt-Universität zu Berlin, and Berlin Institute of Health, Berlin, Germany; 4 DKMS Clinical Trials Unit, Dresden, Germany; 5 Institut für Klinische Chemie und Laboratoriumsmedizin, Universitätsklinikum Carl Gustav Carus an der TU Dresden, Dresden, Germany; 6 Nationales Centrum für Tumorerkrankungen (NCT), Partnerstandort Dresden, Dresden, Germany

**Keywords:** Medical research, Cancer

## Abstract

The accumulation of somatic mutations in hematopoietic stem cells during aging, leading to clonal expansion, is linked to a higher risk of cardiovascular mortality and hematologic malignancies. Clinically, clonal hematopoiesis is associated with a pro-inflammatory phenotype of hematopoietic cells and their progeny, inflammatory conditions and a poor outcome for patients with hematologic neoplasms and solid tumors. Here, we review the relevance and complications of clonal hematopoiesis for the treatment of hematologic malignancies with cell therapeutic approaches. In autologous and allogeneic hematopoietic stem cell transplantation native hematopoietic and immune effector cells of clonal origin are transferred, which may affect outcome of the procedure. In chimeric antigen receptor modified T-cell therapy, the effectiveness may be altered by preexisting somatic mutations in genetically modified effector cells or by unmodified bystander cells harboring clonal hematopoiesis. Registry studies and carefully designed prospective trials will be required to assess the relative roles of donor- and recipient-derived individual clonal events for autologous and allogeneic cell therapies and to incorporate novel insights into therapeutic strategies.

## Introduction

Somatic mutations accumulate in aged tissues and may contribute to malignant transformation [1–8]. Most of the work so far has focused on genomic alterations in exons, however mutations are also observed in non-coding regions [9]. Common alterations are single base exchanges and short insertions and deletion (indels) resulting from error-prone repair mechanisms. In addition to genetic changes, malignant transformation is fueled by epigenetic modifications, such as altered expression patterns of genes involved in tumorigenesis like oncogenes and tumor-suppressor genes [10,11]. Signs of aging are further the increased frequency of other mutational events including larger chromosome breaks, that accumulate throughout the life span of a tissue, like hematopoiesis [12–17].

**Fig. 1:**
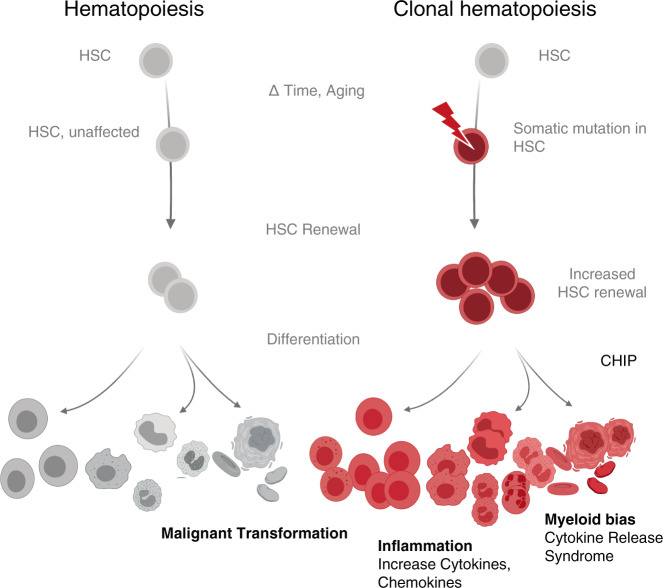
Contrasting hematopoiesis with clonal hematopoiesis. CH, which describes the accumulation of somatic mutations through aging and stress over an individual’s lifetime, may result in clonal hematopoiesis of indeterminate potential, CHIP. Various pathological conditions have been associated with CH dominance. Pictograms: BioRender.com.

In hematopoiesis, the clonal dominance of a subset of hematopoietic stem and progenitor cells (HSPC) and their progeny, as detectable by patterns of somatic mutations, is termed clonal hematopoiesis (CH, Fig. 1). Besides aging, also smoking and germline genetic variants are associated with emerging CH and CH could even be observed in newborns [9,18–20]. Therefore, varying factors influence the frequency of CH in a given cohort, which is additionally influenced by the sensitivity of the applied detection method. Whereas targeted deep sequencing of specific gene regions may detect 1/1000 mutated alleles in a given sample, whole genome sequencing typically detects VAF of 2–5% [9,21,22].

CH is not restricted to malignant conditions like acute myeloid leukemia (AML) and myelodysplastic syndrome (MDS) but can be also observed in ostensibly healthy persons. Indeed, the terms age-related clonal hematopoiesis and clonal hematopoiesis of indeterminate potential (CHIP) have been introduced to describe the occurrence of somatic mutations in healthy persons without hematologic abnormalities [7,23]. CHIP is defined as the manifestation of cancer-associated somatic driver-mutations with a variant allele frequency (VAF) of a least 0.02 (corresponding to roughly 4% of cells for heterozygous mutations) [23]. The prevalence of CHIP is also dependent on age, while about 20% of people aged 70 and older harbor CHIP, this affects <1% of the population below 40.

Exon alterations in CH are restricted to a well-defined set of genes. Two-thirds of clonal events associated with CHIP are dominant-negative or loss-of-function mutations in *DNMT3A*, *TET2*, and *ASXL1*, all of them being epigenetic regulators. Other recurring mutations observed in CHIP most frequently relate to DNA damage response (DDR) (e.g., *TP53*, *PPM1D*), growth factor signaling (e.g., *JAK2*, *CBL*) and components of the spliceosome machinery (e.g., *SF3B1*, *U2AF1*). Some of these mutations have been detected in HSPC and provide increased self-renewal, enhanced repopulating activity and reduced differentiation capacity [24–41]. Extrinsic factors, however, further influence the fate of mutated HSPC. For example, mutations in DDR genes like *PPM1D* confer survival advantage under repetitive cytotoxic conditions [42,43]. Other mechanisms that propagate clonal progression are mostly unknown, but first functional studies in preclinical models suggest an increased activity of inflammatory circuits being involved in CHIP progression [44].

The transition of somatic mutations from HSPC to all mature lineages may be expected. In experimental models and in human samples with high allele frequency (typically ≥ 0.02 VAF), mutational burden was instead highest in cells of the myeloid lineage, indicative for a preferential myeloid differentiation, termed myeloid skewing/bias [29,36,38,45–48]. By using more sensitive assays it was observed that in samples with low allele frequency (<0.02 VAF) the mutational burden was equally distributed between different lineages [21]. As the mutational spectrum differs between high and low mutational burden samples, myeloid skewing probably does not represent a phenomenon of CH in general, instead it could reflect a feature of clones acquiring dominance at later time points, potentially due to pro-inflammatory cytokine loops [44,49]. Despite myeloid skewing and risk of transformation to myeloid neoplasms (MDS/AML; low in absolute numbers) being common, CHIP should not be regarded as a disorder restricted to the myeloid lineage but has pleiotropic consequences. First, in individuals with CHIP the incidence of lymphatic neoplasms is also elevated but the pathophysiological link to CH is less obvious. In contrast to myeloid neoplasms, these lymphomas do not necessarily arise out of mutated HSPC by stepwise acquisition of additional mutations [50–54]. Second, CH enhances the risk of inflammatory diseases like cardiovascular disease (CVD) [7,18,55,56] and adult onset autoinflammatory disease [57]. CH patients are described to be enriched in illnesses like hemophagocytic lymphohistiocytosis [58], severe COVID-19 [59], anti-neutrophil antibody-associated vasculitis [60], to name a few examples of the expanding list. Third, cytostatic therapy, but not immune checkpoint blockade, is a major risk factor for development of CH and shapes the mutational spectrum [15,61–63]. Conversely, the presence of CH negatively impacts the prognosis of patients with solid cancers [62] and aggressive lymphomas [64] undergoing cytostatic therapy. Although the mechanistic link between CH and this large number of various disorders remains to be elucidated, CH-associated inflammation appears as the overarching principle. Diverse mediators of innate and adaptive immunity have been described to be modulated by the occurrence of specific CH-associated somatic mutations [65,66]. Not only cellular components of the myeloid lineage like monocytes/macrophages [56,67–70], mast cells [71], neutrophils [72], but also lymphatic cells [73–77] can exert altered function.

**Fig. 2:**
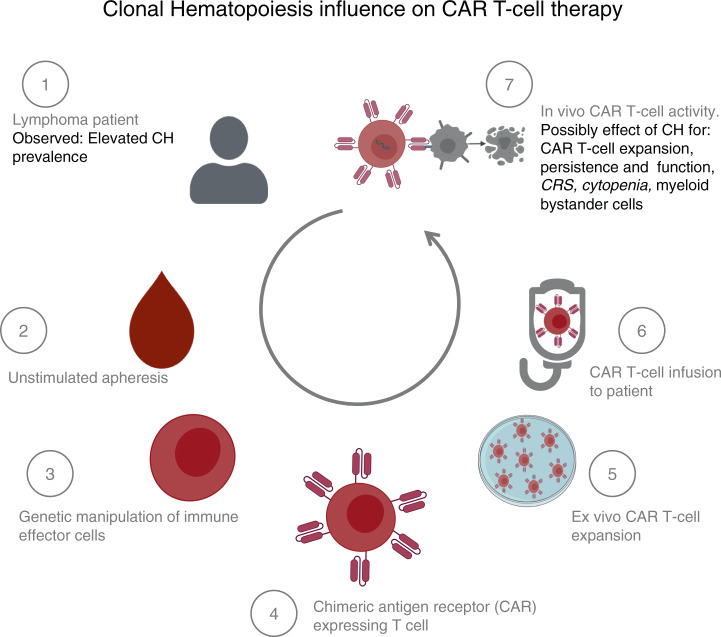
Conceivable effects of clonal hematopoiesis on CAR T-cell therapy. CH might theoretically impact CAR T-cell processing and treatment (gray text) at all stages. Already observed effects attributed to CH (black text). Pictograms: BioRender.com.

In view of the plethora of consequences of CH, all aspects of cellular therapies, from cell harvest to cell processing and product/graft function (Fig. 2 showing exemplary CAR T-cell therapy) might be affected. The purpose of this review is to (1) summarize the available information on the clinical relevance of CH in the context of both hematopoietic cell transplantation (HCT) as well as the application of genetically engineered T cells, and to (2) provide some insights into published preclinical information, which may stimulate ongoing and future translational research studies.

## Clonal hematopoiesis and autologous hematopoietic cell transplantation

Somatic mutations associated with myeloid malignancies (≥2% VAF) can be detected in 5–30% of specimen from unselected, heavily pretreated patients undergoing autologous transplantation [77–80]. Again, the frequency of CH also depends on the sensitivity of the applied method. Even in less intensively treated patients undergoing autologous HCT, the prevalence of CH is 11–22% when lowering the VAF threshold to 0.01 and 0.02, respectively [81]. In summary, the prevalence of CH in patients undergoing autologous HCT is elevated due to previous exposure to conditions that promote CH (e.g., chemotherapy) when compared to epidemiological studies in hematological healthy persons.

Autologous grafts are predominantly obtained by chemotherapy combined with G-CSF, or G-CSF alone (steady state mobilization). In poor mobilizers, G-CSF is combined with plerixafor to increase HSC yield. There is no systematic data available in humans how these various mobilization strategies differentially influence graft composition with regard to the proportion of contained clonal cells. For example, apheresis following chemotherapy might theoretically enrich for HSC bearing mutations in DDR genes. More important, the presence of CH has been suggested to affect process-related results and to influence short- and long-term clinical outcome parameters. To this end, the presence of CH has been associated with inferior mobilization capacity [76,80–83] and delayed engraftment [84], however, inferior mobilization is not a consistent finding [79,84].

**Fig. 3:**
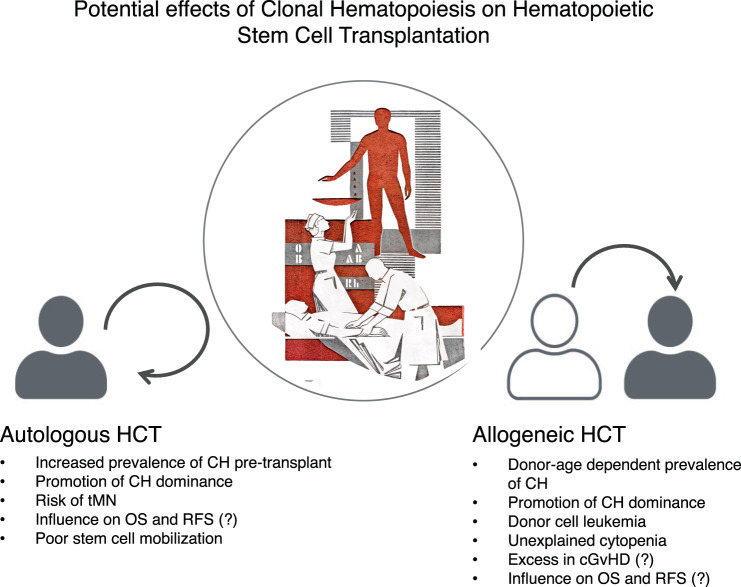
Impact of clonal hematopoiesis on outcome of hematopoietic cell transplantation. The presence of CH in the graft has been associated with various findings in allogeneic, as well as autologous hematopoietic stem cell transplantation. The potential influence of different mobilization procedures on CH recovery within the graft is not depicted. Image showing blood transfusion: scraffitto at the University Hospital Dresden by Alfred Hesse, photo: T. Albrecht.

The presence of CH in the autologous graft has in a few retrospective studies been linked to an increased risk for therapy-related myeloid neoplasms (tMN) [78,80,83] and reduced overall survival (OS) [80,81], partly due to an increase in CVD [80]. Assuming that the detection of somatic mutations in the autologous graft simply reflects the presence of CH, the increase in myeloid neoplasms and CVD-associated mortality, when compared to non-CH patients, merely confirms the known risk profile. It remains unclear whether the mobilization process itself (e.g., by enrichment of CH clones) can accelerate the progression of CH-associated diseases and the increase in non-relapse mortality (NRM). Future research should investigate whether purging of CH from the graft might have positive effects on the overall outcome after autologous HCT. Longitudinal tracking of somatic mutations in patients receiving autologous HCT revealed that impressive changes (increase in VAF and a gain in new mutations) could only be observed in temporal proximity to autologous HCT. In subsequent peripheral blood (pB) samples (without continued chemotherapy), mutational burden remained mostly stable [43,84]. Therefore, hematopoietic stress might help CH to dominate wild-type hematopoiesis and might accelerate CH-associated non-hematologic diseases. However, in other studies no abrupt and only modest changes in mutational burden directly after autologous HCT was described [76,77]. Remarkably, presence of CH in the context of autologous HCT did not necessarily translate into decreased OS or excess in NRM [43,79]. In patients with MRD negative MCL after first-line therapy, four patients developed tMN. Only one of them was CH positive at the time point of transplantation [43]. Also in MM, presence of CHIP at autologous transplantation was not predictive for tMN [81]. In lymphoma patients, an increase in tMN was not associated with bulk CH, but with mutation in DDR genes [78] (Fig. 3).

The cumulative incidence of relapse usually did not differ between CH and non-CH patients, suggesting that presence of CH did not influence the therapeutic efficacy of autologous transplantation [43,80].

In summary, the prevalence of CH at the time point of autologous transplantation mainly depends on age and pretreatment intensity. Autologous HCT, like other hematopoietic stressors, represents a risk factor for CH. The contribution of various procedures (e.g., graft, mobilization, conditioning regimen) however remains vague and the impact of CH on outcome may vary between different cohorts, likely depending on continued treatment, affected genes, and mutational burden. To date, the effectivity of autologous HCT for the underlying disease seems unaffected by the presence of CH. Therefore, the presence of CH should currently not impact on clinical decision-making in the context of autologous HCT.

## Clonal hematopoiesis and allogeneic hematopoietic cell transplantation

Donor cell-derived myeloid malignancies, summarized as donor cell leukemia (DCL), include AML and MDS and occur after allogeneic HCT. It is conceivable that DCL may be promoted by transplantation of grafts that are burdened by CH (Fig. 3). In a European survey, the frequency of DCL was 0.8%. In 28% of evaluable patients, transplanted CH could be retrospectively identified [85]. Rare cases of simultaneous development of myeloid neoplasms in donor and recipient, originating from the same clone, further underscore transplantability of preleukemic clones [86].

In contrast to autologous HCT, where grafts are harvested from the aged patient, the unrelated donors in allogeneic HCT are usually younger [87]. These younger donors were less exposed to hematopoietic, CH-boosting stressors, the expected CHIP (≥0.02 VAF) prevalence is therefore <1% [6–8]. However transplantation and GvHD have been suggested to accelerate aging of transplanted donor-derived HSPC [88], which in turn could foster emergence of CH.

Sibling donors are often older than unrelated donors, consequently in grafts from sibling donors ≥55 years CHIP prevalence was 16% [89]. After allogeneic HCT, unexplained cytopenia has been associated with the presence of donor-engrafted CHIP and indeed the donors with CHIP were older and predominantly siblings [90]. In the most comprehensive study so far, presence of CHIP in the graft of donors ≥55 years was a risk factor for emergence of DCL. It has been shown that almost all donor-derived CHIP successfully engrafted the recipients. CHIP-transplanted patients showed an increase in the incidence of chronic graft versus host disease (cGvHD), but not in cGvHD severity and also not in the incidence of acute GvHD. Remarkably, increase in cGvHD was attributable to a significant excess of ocular GvHD, while skin and visceral cGvHD were equally distributed between both cohorts. This organ tropism is so far unexplained and leaves room for different hypotheses. In the CHIP positive cohort, a numerical, but not statistically significant rise in NRM could also be observed. For the entire population, donor CHIP was associated with a significantly reduced cumulative incidence of relapse/progression (CIR/P), but this observation was mainly attributable to patients being not in complete remission (non-CR) before allogeneic HCT. Finally, OS of the entire population was not influenced by the presence of CHIP in the donor. Again only in the subpopulation of non-CR patients, presence of CHIP in the donor was associated with preferable OS [89]. In another study with donors ≥40 years, the presence of *DNMT3A* mutations (but not other mutations) in the graft with ≥0.01 VAF was associated with improved progression-free survival in a large heterogenous cohort of patients. The association even remained statistically significant in a multivariable model. The effect was driven by patients who did not receive posttransplant cyclophosphamide. These patients showed a reduced relapse risk and an increase in cGvHD [91]. Further results are expected, as this study is not fully published yet.

To sum up, donor CH, specifically donor CHIP, is a risk factor for DCL (Fig. 3). Because of the low incidence of DCL, a systemic screening of the entire donor cohort will probably not substantially influence OS of the entire recipient population. In selected cases (older donors and several alternative donors available) CH-screening might help in the donor selection process. This might be of special interest in related donors, as the incidence of CHIP has been described to be elevated in relatives of patients with myeloid neoplasms [89]. First retrospective analyses suggest a potential impact of donor CHIP on relapse incidence and chronic GvHD [89], however further confirmatory analyses in independent cohorts are required.

## Clonal hematopoiesis and genetically engineered autologous T cells

The vast majority of immune effector cells (IEC) for genetic modification (e.g., chimeric antigen receptor CAR T cells) are extracted from pB samples after unstimulated leukapheresis. Clinical grade IEC are furthermore subject to a variety of procedures including viral transduction and expansion of the requested population in vitro (Fig. 2). Somatic mutation associated with CHIP might directly (mutation in transduced T cells) and indirectly (mutation in bystander cells) affect IEC activity.

To date, clinical data about the interference of CH with CAR T-cell therapy (CART) is still scarce. Circumstantial evidences however suggest that CH might influence CART toxicity as well as effectivity. Treatment with CAR T cells is frequently complicated by the cytokine release syndrome (CRS). Recipient myeloid cells play a critical role in CRS development. Monocyte/macrophage-derived IL-1 and IL-6 were demonstrated to orchestrate this inflammatory process [92,93]. Presence of CH in men has been associated with various inflammatory conditions and in *TET2*-mutant mice monocytes/macrophages showed pro-inflammatory characteristics and substantially contributed to progression of arteriosclerotic disease by secretion of IL-1 and IL-6 [56,67]. Thus, it appears possible that patients with CH have an increased risk for inflammatory side effects of CART. Larger cohort studies investigating such associations are pending.

Patients undergoing CART have been heavily pretreated and therefore have an elevated risk for the presence of CH. Although CH frequency in CART patients has not been systematically reported, we expect a CHIP prevalence at least as high as in lymphoma patients undergoing autologous HCT. Sterile inflammation (clinically recognized as CRS) is a prototypic side-effect of CART. Under clinical aspects, CRS is very limited in time, but it cannot be excluded that this inflammatory episode is sufficient to push clonal progression and to increase the risk of tMN. Furthermore, prolonged cytopenia is a frequent observation after CART. Etiology of delayed hematopoietic recovery remains vague but it has been associated with occurrence of severe CRS [94]. In autologous HCT, presence of CH was linked to a delay in engraftment [84]. Thus, it is conceivable that presence of CH negatively affects hematological regeneration. Again, clinical data that underpin these hypotheses are missing.

Some genes frequently mutated in CH also play a pivotal role in regulation of lymphocyte activity and might influence clinical outcome parameters (toxicity, effectivity) of CAR T cells. *TET*-family of genes are critical for the development and function of various lymphocyte subset. Homozygous loss of function of *TET*-genes has been shown to promote autoinflammation [95,96] and to alter antigen-specific responses [74]. Accordingly, homozygous loss-of-function mutations of *TET2* can lead to changes in activity of human T-cell subsets genetically modified to express CAR. One patient with chronic lymphocytic leukemia, 2 months after a second infusion of anti-CD19 CAR T cells with CD28 co-stimulation domain, developed an atypical, delayed expansion of monoclonal CD8^+^ CAR T cells with central memory phenotype, which was associated with emergence of CRS. The CAR T-cell population showed long-term persistence and the patient experienced sustained clinical response. These monoclonal CD8^+^ CAR T cells were characterized by compound heterozygous loss-of-function of *TET2*. One *TET2* allele was disrupted by CAR lentiviral integration, the second allele by a E1879Q mutation, which was present also in non-CAR hematopoietic cells therefore representing CH. After CAR-specific stimulation, CAR T cells with compound heterozygous *TET2*-mutation showed an increased expression of various cytokines including INF-γ compared to CAR T cells with lentiviral integration sites outside *TET2*. Compound heterozygous *TET2*-mutated CAR T cells were less prone to senescence and showed prolonged activation capacity after repetitive CAR-specific stimulation [97]. This example suggests that both CAR integration site [98] and somatic mutations in the context of CH might directly and indirectly influence CAR T-cell activity and clinical outcome.

## Summary and conclusion

Several observations indicate that CH might impact most cellular therapies currently established for patients with hematologic malignancies. In patients undergoing autologous HCT, it remains to be elucidated precisely how the presence of CH impacts on clinical outcomes. Manipulation of CH in the graft could represent a therapeutic approach, for example by targeted inhibition of pathways driven by somatic mutations [99]. In the context of allogeneic HCT, donor testing for CH has not been included in routine diagnostic procedures. Despite emerging evidences toward an impact of donor CH on transplant outcome, the long-term clinical consequence of the transfer of grafts with CH is so far less well-defined. Future studies will have to address whether CHIP should be integrated in donor selection algorithms. Patients with detectable CHIP after autologous or allogeneic HCT may be at an increased risk for tMN and monitoring strategies are warranted. In CART, the impact of CH on clinical outcome (toxicity and efficacy) has so far not been investigated, but first evidences for interaction are emerging. Purging of autologous CAR T-cell products and usage of allogeneic CAR T-cell products would offer therapeutic options. More importantly, harnessing the epigenetic effects of certain mutations involved in CH to improve the efficacy of CAR T cells is an exciting area of ongoing research. In summary, while detailed studies are necessary and critical, it is apparent that considering CH status of patients may further pave the way toward effective and patient-centered, personalized treatment regimes.
